# Buoyancy studies in natural communities of square gas-vacuolate archaea in saltern crystallizer ponds

**DOI:** 10.1186/1746-1448-2-4

**Published:** 2006-04-14

**Authors:** Aharon Oren, Nuphar Pri-El, Orr Shapiro, Nachshon Siboni

**Affiliations:** 1 Department of Plant and Environmental Sciences, The Institute of Life Sciences and the Moshe Shilo Minerva Center for Marine Biogeochemistry, The Hebrew University of Jerusalem, 91904 Jerusalem, Israel; 2 Department of Environmental Hydrology & Microbiology, Zuckerberg Institute for Water Research, the Jacob Blaustein Institute for Desert Research, Ben-Gurion University of the Negev, Sede Boqer Campus 84990, Israel; 3 Department of Biotechnology Engineering, Ben-Gurion University of the Negev, Beer-Sheva 84105, Israel

## Abstract

**Background:**

Possession of gas vesicles is generally considered to be advantageous to halophilic archaea: the vesicles are assumed to enable the cells to float, and thus reach high oxygen concentrations at the surface of the brine.

**Results:**

We studied the possible ecological advantage of gas vesicles in a dense community of flat square extremely halophilic archaea in the saltern crystallizer ponds of Eilat, Israel. We found that in this environment, the cells' content of gas vesicles was insufficient to provide positive buoyancy. Instead, sinking/floating velocities were too low to permit vertical redistribution.

**Conclusion:**

The hypothesis that the gas vesicles enable the square archaea to float to the surface of the brines in which they live was not supported by experimental evidence. Presence of the vesicles, which are mainly located close to the cell periphery, may provide an advantage as they may aid the cells to position themselves parallel to the surface, thereby increasing the efficiency of light harvesting by the retinal pigments in the membrane.

## Background

Since the presence of gas vesicles was first recognized in "Bacterium halobium", now *Halobacterium salinarum*, by Helena Petter [1,2], gas vesicles have become beloved study objects in the biology of halophilic archaea of the family *Halobacteriaceae *– from the beautiful electron microscopical stereopictures taken as early as 1956 by Houwink [3] to the in-depth analysis of genes involved in the synthesis of gas vesicles and its regulation [4-10].

As early as 1932 Petter [2] suggested that the presence of gas vesicles and the buoyancy the vesicles bestow upon the cells can be of considerable ecological advantage: the vesicles may buoy the cells to the surfaces of brine pools and salt lakes, where they would benefit from higher concentrations of oxygen, which may easily become a limiting fact in view of its low solubility in highly saline brines. This interpretation was adopted by other workers as well [11,12]. The discovery that light can be used as an energy source by *Halobacterium*, mediated by the retinal protein bacteriorhodopsin [13-15], suggested another possible advantage for halophilic archaeal cells to float: an increase in the available light intensity. A third possible function suggested for the gas vesicles, namely their use as light-shielding organelles to protect the organism exposed to high light intensities in its environment against harmful ultraviolet radiation, was not supported by controlled laboratory experiments [12].

In spite of these theoretical advantages of the possession of gas vesicles for a halophilic archaeon in hypersaline water bodies, we have not found any reports in the literature confirming that indeed such cells do float toward the surface in their natural environments [7,8,16,17]. Moreover, gas vesicles are not widely distributed among the halophilic archaea. Of the more than 50 described species of *Halobacteriaceae*, only four have been shown to produce them: *Halobacterium salinarum*, *Haloferax mediterranei*, *Halorubrum vacuolatum*, and *Halogeometricum borinquense *[17], and these species are by no means the dominant ones in hypersaline lakes, salterns, and other aquatic hypersaline environments. We are not aware of the existence of any environment in which any of these four species occurs in sufficient numbers to enable ecological studies.

The only gas-vacuolate halophilic archaeon known to be present in high numbers in certain hypersaline environments is the square flat organism found by Walsby in numbers as high as 7 × 10^7 ^cells per ml of brine in a coastal salt pool on the Sinai peninsula, Egypt [18-21]. This type of organism, previously overlooked, was then rapidly recognized to be one of the most abundant members of the microbial community in saltern crystallizer ponds worldwide (Spain, Israel, Mexico, Australia, and elsewhere) [17,22-30], and in certain natural salt lakes as well [31]. The square archaea were reported to represent at least 55% of the total numbers of prokaryotes in the salterns of Eilat, Israel [25], and 48–61% and 22–32% in the salterns near Alicante and Tarragona, Spain, respectively [22,23]. These unusual archaea were only recently brought into culture [20,30,32,33], and a formal description of the species as *Haloquadratum walsbyi *is to be expected soon.

The presence of dense populations of the square gas-vacuolate cells in saltern crystallizer ponds presents us with a unique opportunity to study the possible role of the gas vesicles in the life of halophilic archaea in their natural habitat. Here we report our attempts to assess the degree of buoyancy provided by the gas vesicles in the community of square archaea in the saltern crystallizer ponds of Eilat, Israel.

## Results

### Chemical and biological properties of the brine samples

The crystallizer brine samples used in this study contained between 356–373 g l^-1 ^salt and had densities between 1227 and 1238 kg m^-3^. The brine samples were pink because of the large numbers of β-carotene-rich *Dunaliella *cells (190–1700 cells/ml) and 2.4–3.6 × 10^7 ^prokaryotic cells ml^-1 ^(Table 1). These numbers are similar to those given earlier for the Eilat crystallizer ponds: between 8.9 × 10^6 ^and 4.3 × 10^7 ^cells ml^-1 ^were reported in 1993 and 1995 [25,26]. Flat square and rectangular gas-vesicle containing archaea dominated the prokaryote community: between 70 and 81% of all cells had this morphology, values higher than the 20–23% and 55% reported in earlier years [25,26]. All these values may be underestimations as a flat square or rectangular cell positioned perpendicular to the plane of the microscope slide can easily be mistaken for rod-shaped prokaryotes.

**Table 1:** Chemical and biological properties of the brine samples examined.

Sample	Date	Pond no.	Density (kg m^-3^)	Prokaryotes (cells ml^-1^)	Relative abundance of square cells (%)
1	April 6, 2005	304^a^	1,238 (25°C)	2.4 ± 0.3 × 10^7^	70
2	June 8, 2005	305^b^	1,227 (29°C)	3.6 ± 0.3 × 10^7^	80
3	August 30, 2005	301	1,240 (33°C)	2.6 ± 0.4 × 10^7^	81

^a ^For a map of the Eilat saltern ponds and the location of the ponds, see [34].

^b ^Pond 305 is the former evaporation pond 205, in recent years converted to a crystallizer pond.

**Figure 1:**
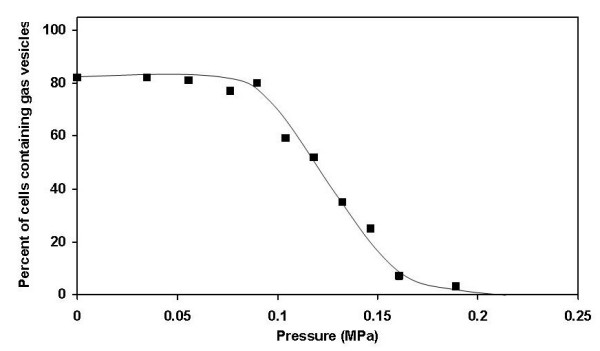
Pressure collapse curves of square archaea from sample 3. The plot shows the percentage of the flat square/rectangular cells showing presence of refractile gas vesicles following exposure to increasing pressure.

To test the pressure sensitivity of the gas vesicles of the square archaea, brine samples were exposed to increasing levels of pressure and the number of square cells that still contained microscopically recognizable gas vesicles, showing as bright intracellular inclusions when viewed with phase contrast optics, was assessed. Most cells lost their gas vesicles at pressures between 0.1 and 0.15 MPa, half of the cells having lost their refractile gas vesicles at 0.126 MPa (Fig. 1). Walsby [18] reported that in the square archaea he collected from the brine pool in the Sinai peninsula, a definite reduction in gas vesicle numbers per cell was seen after application of 1.5 bars [0.15 MPa], that most of the vesicles had disappeared by 2.5 bars [0.25 MPa], and that none remained beyond 3 bars [0.3 MPa]. In a similar experiment with *Halobacterium *NRC-1, a pressure of 0.14 MPa was required to cause loss of refractile gas vesicles in 50% of the cells (not shown). These values are higher than the mean critical collapse pressures of the individual gas vesicles, as each cell contains vesicles of different strength, so that a cell in which more than half of the vesicles had been collapsed still shows refractile vesicles in the phase contrast microscope. For *Halobacterium *NRC-1 we determined a mean critical collapse pressure of 0.091 MPa, based on optical density measurements of cell suspensions (not shown). No parallel measurements could be performed with the square archaea in Eilat brine samples as the sensitivity of the turbidity assay was insufficient for the cell density in the brines.

### Assessment of the vertical movements of square halophilic archaea in saltern crystallizer brine

When samples of brine were placed in a Petroff-Hauser counting chamber (depth 20 μm) and left to stand for up to 4 hours, no accumulation of square cells near the coverslip could be observed. Instead, cells remained distributed evenly within the space between slide and coverslip. However, when the brine sample was first subjected to pressurization, causing collapse of all gas vesicles, most cells sank to the bottom of the 20 μm "water column".

**Figure 2:**
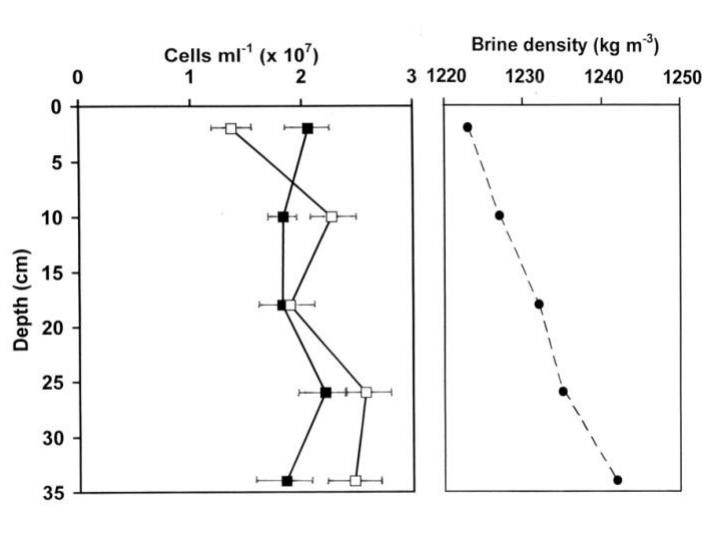
The vertical distribution of prokaryotic cells in brine sample 3 after 60 h (closed squares) and 110 h of incubation (open circles) indoors in a 1 liter glass cylinder in a density (salt) gradient.

Another approach used to assess vertical movement of cells in the brine was to incubate brine in 1 liter glass cylinders, while introducing a slight salinity gradent to prevent mixing of the contents by convection currents [35]. In this experiment, performed with brine sample no. 3 only (Table 1), little change in vertical distribution of the archaeal cells could be demonstrated after up to 4.5 days, except for a tendency for a small decrease of cell numbers in the upper layer toward the end of the incubation period (Fig. 2). *Dunaliella *cells tended to accumulate at the meniscus as a result of their low density (due to the use of glycerol rather than KCl as osmotic solute, as well as to their massive accumulation of β-carotene granules), and by active flagellar motility, swimming toward the light.

**Figure 3:**
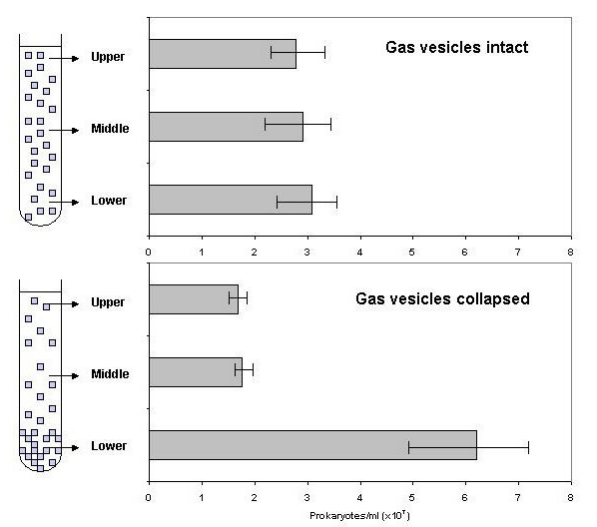
Vertical distribution of prokaryotes from Eilat brine sample 1 (upper panel) and an identical sample in which the gas vesicles had been collapsed by pressurization (lower panel), following centrifugation for 12 h in 10 ml portions at 500 rpm in a SS-34 rotor (radius of centrifugation: 9.4 cm).

To further investigate the potential of the square archaea to float towards the surface of the saltern brines, "accelerated flotation" experiments were performed in which brine samples from all three sampling dates were centrifuged at speeds insufficient to cause collapse of the gas vesicles. Cells were still homogeneously suspended after 12 h centrifugation at 26 × *g *at the bottom of the tubes (Fig. 3). This centrifugation is equivalent to incubation for 13 days at normal gravity, as according to Stokes's equation (see below), the sinking velocity is proportional to the acceleration due to gravity. A similar result was obtained following 12 and even 60 h centrifugation in a swing-out rotor at 39.1 × *g *(results not shown). Taking into account the depth of the brine in the tubes (5.6 cm and 2.9 cm, respectively), its density, and the angle or the rotor, the calculated maximum pressure exerted on the cells was 0.010 and 0.014 MPa, respectively, insufficient to cause collapse of a significant fraction of the gas vesicles (compare Fig. 1).

## Discussion

The results presented above suggest that the gas vesicles present in the square halophilic archaea in the saltern ponds of Eilat provide negligible floating velocity to the cells. The general assumption that the gas vesicles enable the square archaea to float to the surface of the brine in which they live, resulting in an ecological advantage, was therefore not supported by our experimental evidence. Our observations with field-collected material are supported by the behavior of the gas-vacuolate square archaea in culture. In static culture, most cells of the strain isolated by Burns et al. [30] do not float but remain homogenously dispersed (our results, and M.L. Dyall-Smith, personal communication); only a small population of relatively large cells floats after sufficient time. Effective flotation of cells can be observed in cultures of *Halobacterium salinarum*, but this organism was never shown to dominate in any aquatic hypersaline habitat.

Walsby's observations suggested that in the Sinai brine pool, in which the square archaea were first discovered, the gas vesicles indeed provided positive buoyancy. Thus, Walsby [18] enriched the square archaea by accelerated flotation by low speed centrifugation, generating pressures not exceeding 0.04 MPa. He also found the square cells to aggregate at the meniscus of the brine sample after leaving it to stand for two days. A combination of static incubation for 3 days and low speed centrifugation was used by Parkes and Walsby [19] to concentrate the cells used in their electron microscopy studies. Prolonged incubation without shaking was also used by Kessel and Cohen [21] to collect material; however, the final cell density reported was only approximately 50 square cells per ml.

When discussing the energetic cost and the ecological profit of the gas vesicles [36], it should be taken into account that in aquatic environments such as salt lakes and salterns, considerable mixing occurs by wind and waves. Accordingly, even when during static incubation under laboratory conditions some cells may float toward the surface, this does not yet imply that such cells will also be buoyed up under natural conditions. Gas vesicle-containing cyanobacteria such as *Microcystis*, *Anabaena*, *Trichodesmium *and others can form surface blooms because they have large cells, often with a very high content of gas vesicles, so that the difference in density between the cells and their medium is substantial. Furthermore, they grow in colonies, filaments or bundles of filaments (increasing their effective radius in Stokes's equation; see below), and they live in a medium of half the viscosity of that of the halophilic archaea. Some tendency has been reported of the square archaea to form small sheets when cells do not separate after division. Kessel and Cohen [21] documented such a sheet of 64 cells, and they reported that sheets of eight cells were not uncommon in the Sinai brine pool. Similar observations of small groups of cells remaining attached can be found elsewhere [18,23,37], and such behavior can of course increase the efficiency of flotation of the cells (see also the calculation in Table 2).

**Table 2:** Theoretical calculated rates of flotation of square archaea in saltern brines, based on different values for cell dimensions, the nominal radius [(length × width × height)^1/3^], the K value (the ratio of the velocity of a sphere with a radius equal to the nominal radius of the particle and the velocity of the particle), as calculated according to algorithms given by McNown and Malaika [39], and the difference in density between the brine and the cells. The "cell" with dimensions 24 × 24 × 0.15 μm represents a sheet of 8 × 8 3 μm-wide cells, such as observed occasionally in brine pools [21].

Cell dimensions (μm)	Nominal radius (μm)	K value	(ρ_1 _– ρ_2_) (kg m^-3^)	Flotation rate	
				(m s^-1^)	(mm day^-1^)
2 × 2 × 0.2	0.98	1.98	-30	15.8 × 10^-9^	1.37
2.5 × 2.5 × 0.2	1.08	1.84	-30	20.1 × 10^-9^	1.80
3 × 3 × 0.15	1.11	2.31	-30	17.4 × 10^-9^	1.51
24 × 24 × 0.15	4.42	4.61	-30	138 × 10^-9^	11.9
2 × 2 × 0.2	0.98	1.98	-130	68.7 × 10^-9^	5.9
2.5 × 2.5 × 0.2	1.08	1.84	-130	89.7 × 10^-9^	7.8
3 × 3 × 0.15	1.11	2.31	-130	75.5 × 10^-9^	6.5
24 × 24 × 0.15	4.42	4.61	-130	600 × 10^-9^	51.8

When estimating the rate of sinking or flotation of a prokaryotic cell in the water column, the first approximation is that determined by Stokes's equation for a spherical particle [38]:

where

V = velocity of fall,

g = acceleration of gravity,

r = "equivalent" radius of particle,

ρ_1 _= density of particle,

ρ_2 _= density of medium,

η = dynamic viscosity of medium.

To estimate the floating or sinking speed of square archaea in saltern brines, we may use the following values of the different parameters:

g – constant at 9.8 m s^-2^.

r – A typical square archaeon of 2.5 × 2.5 × 0.2 μm will have a volume of 1.25 μm^3^. A sphere with the same volume will have a radius of 0.67 μm. However, the hydrodynamic behavior of the square cells cannot be simulated by a sphere of the same volume, as a flat particle will encounter increased drag forces when moving in the water column with the direction of motion parallel to the short axis of that particle. For the flat square cells, the effect of particle shape can be estimated from formula 5 presented in Table 1 in McNown and Malaika [39]. For a particle of the above-mentioned dimensions, the shape increases the effective Stokes radius by a factor of 1.98; values in the same order of magnitude can be calculated for differently sized squares (see Table 2).

(ρ_1 _– ρ_2_) – the difference in density between the brine (1,230 kg m^-3^) and the cells. While there may be no direct way to assess the density of the square archaeal cells, we can to some extent predict the value based on our understanding of their anatomy and the density of their components. Protein has a density of about 1,330 kg m^-3^. Nucleic acid is heavier (>1,660 kg m^-3^), while glycolipids are light (1,050 kg m^-3^). It also should be taken into account that the cytoplasmic components are suspended in a saturated solution of KCl (density of 1,182 kg m^-3^). The special geometry of the cells, with a large surface of (glyco)protein cell wall and a relatively small volume of cytoplasm, has also important implications when attempting to calculate the density of the cells. From the electron micrographs published by Stoeckenius [37] we estimate that the cell envelope occupies about one third of the total volume of the cell. When assuming that such electron micrographs reproduce the true dimensions of the cells and their components rather than fixation artifacts, and when further assuming an overall density of the envelope, including the lipid component, of 1,300 kg m^-3 ^and a cytoplasm of 5% nucleic acid, 15% protein and 80% saturated KCl solution, we obtain an estimated density of the cells of 1,250 kg m^-3^, just slightly higher than the density of the brine in which they grew (1,227 to 1,238 kg m^-3^; Table 1). Granules of the storage polymer poly-β-hydroxybutyrate (PHB), often found inside the cells [18,21,37], will not have a great impact on the overall density of the cell (reported density of PHB: 1,285 ± 30 kg m^-3 ^[40,41].

η – the dynamic viscosity of the brine: here we will use a value of 2 × 10^-3 ^kg m^-1 ^s^-1^, about equivalent to the viscosity of a saturated NaCl solution at 20°C. The dynamic viscosity of a saturated NaCl solution (5.32 M) is 1.986 times that of distilled water [42]. The value for saltern brines is probably somewhat higher due to the presence of other ions as well. For comparison, Dead Sea water of a density of 1,214 kg m^-3 ^which contained 309 kg m^-3 ^salts (164 kg MgCl_2_, 118 kg NaCl, 16.2 kg KCl, 42.7 kg CaCl_2_, and 0.92 kg CaSO_4_) was 2.84 times as viscous as distilled water (J. Lati, The Dead Sea Works, Ltd., personal communication). On the other hand, a temperature increase from 20 to 30°C in summer will cause a 20% decrease in dynamic viscosity [38].

Table 2 presents a theoretical calculation of the rates of flotation to be expected for the square cells in a static water column, for cells with a density of 1,200 kg m^-3 ^[(ρ_1 _– ρ_2_) = 30 kg m^-3^] and also for a more extreme case of more highly buoyant cells with cells of 1,100 kg m^-3 ^[(ρ_1 _– ρ_2_) = 130 kg m^-3^]. To achieve such densities, 4.3, respectively 12.9% of the volume of cells of 1,250 kg m^-3 ^should be occupied by gas vesicles, assuming a density of 90 kg m^-3 ^for these gas vesicles (somewhat higher than the value of 60 kg m^-3 ^reported for the wider gas vesicles of *Halobacterium *but lower than the value of 120 kg m^-3 ^calculated for the narrower vesicles of the cyanobacterium *Anabaena *[16]). The calculated rates, of a few millimeters per day only, suggest that, even when the water column is not subjected to any mixing by waves and currents, the square archaea in the salterns cannot be expected to float toward the surface of the brine at a significant speed. A higher rate of flotation ma be achieved by aggregates of cells (see e.g. [21]), but these were only very seldom encountered in the samples examined.

Based on the above calculations it is obvious that the function of the gas vesicles cannot be the rapid flotation of the square archaea to the surface of the brine. It can also not be the goal of the vesicles to provide neutral buoyancy to prevent the cells from sinking: sinking velocities are minimal also in the absence of gas vesicles, and water turbulence and convection will effectively prevent the settling of the cells. An alternative hypothesis is therefore needed to understand the advantage of the gas vesicles to the cells. The advantage may well be sought in a more effective light harvesting. Indications that the square archaea contain the light-driven proton pump bacteriorhodopsin were already obtained from early studies of material from the Sinai brine pool [43], and this finding was confirmed during the characterization of pure cultures of the isolates obtained [32]. Genome analysis showed two proton pumping bacteriorhodopsins and one chloride pumping halorhodopsin (D. Oesterhelt, personal communication). The gas vesicles are mainly located close to the cell periphery of the cells [19,30]. This may aid the cells to position themselves parallel to the surface, thereby maximizing light absorption by the thin sheets oriented normal to the incoming light [[20]; D. Oesterhelt, personal communication], and making optimally use of photoactive pigments on both sides of the membrane.

## Conclusion

Our experimental data, backed up by theoretical calculations, suggest that the gas vesicles present in the square halophilic archaea that abound in saltern crystallizer ponds in Eilat do not bestow significant positive buoyancy to the cells. Therefore an alternative hypothesis may explain the ecological advantage of the production of gas vesicles: the presence of the vesicles, which are mainly located close to the cell periphery, may aid the cells to position themselves parallel to the surface, thereby increasing the efficiency of light harvesting by the retinal pigments in the membrane. Now that the square archaea have been brought into culture, physiological and genetic experiments can be initiated to study the factors regulating the production of gas vesicles and the degree of buoyancy they confer to these fascinating organisms. Such experiments may eventually lead to a full understanding of the ecological role of the gas vesicles in halophilic archaea.

## Methods

### Brine samples

Samples were taken from the saltern crystallizer brines of the Israel Salt Industries Ltd., Eilat in April, June, and August 2005. Table 1 summarizes the physical and biological properties of the brine samples examined. The density of the brine was determined with the aid of a hydrometer, and the total dissolved salt concentration was assayed by weight after heating known volumes (3–5 ml) to dryness (overnight; 150°C).

Total numbers of prokaryotes were counted microscopically in unstained samples in a Petroff-Hauser counting chamber, using a microscope with phase contrast optics (Nikon, Labophot-2 or Zeiss). When necessary, cells were first concentrated 5–10-fold by centrifugation: portions of 1 ml brine were added to preweighted 1.5 ml plastic centrifuge tubes, the weight was determined again, and after 10 min centrifugation at 13,200 rpm in an Eppendorf 5415 D microcentrifuge, 0.8–0.9 ml of supernatant fluid were withdrawn, the pellet was resuspended in the remaining fluid, and the tubes were weighed again, enabling calculation of the degree of concentration. The relative contribution of square archaea to the total community of prokaryotes was assessed in the phase contrast microscope under a 100× objective. The same microscope setup was also used to assess the percentage of square archaea or *Halobacterium *cells containing refractile gas vesicles after having been exposed to different pressures (see below). A minimum of 100–150 cells was counted for each sample and treatment. For the enumeration of *Dunaliella *cells, 2.5-ml portions of brine were filtered through Millipore SMWP-25 filters (5 μm mean pore size). The orange cells were counted on the filter under a 16× objective. Cell numbers were calculated from the average number of cells per field and the field diameter, calibrated with the aid of the grid of the Petroff-Hauser counting chamber. The relative accuracy of the algal and prokaryotic cell counts was estimated at ± 10 and ± 15%, respectively.

### Halophilic archaea and culture conditions

*Halobacterium *sp. NRC-1 (ATCC 700922) was grown in 250 ml Erlenmeyer flasks with 150 ml portions of medium containing (g l^-1^): NaCl, 250; MgCl_2_·6 H_2_O, 5; KCl, 5, NH_4_Cl, 5, and Bacto yeast extract, 10; pH 7. After 3 days of growth with shaking at 35°C, the flasks were left without shaking at room temperature for 1–2 days. Gas vesicle-rich cells floating near the surface were collected, suspended in salt solution identical of that of the growth medium but without the yeast extract, and used in the experiments as described.

### Pressure-collapse curves

To subject brine samples or *Halobacterium *cultures to different pressures, 2.5 ml portions of brine or *Halobacterium *cell suspensions in glass test tubes were placed in an anaerobic jar converted to a pressurizing vessel by connecting the gassing port in the lid to a nitrogen gas cylinder. The pressure, as monitored with a pressure gauge, was increased to the desired value. The pressure gauge was calibrated by connecting a U-shaped tube filled with mercury to the gassing inlet and recording the height of the mercury column. After pressurizing for at least 30 s the pressure was released, the jar was opened, and the percentage of cells containing refractile gas vesicles estimated microscopically. For *Halobacterium *suspensions the OD_600 _was measured against water, and the percentage of intact gas vesicles was calculated on the basis of the optical density before pressurization (100% vesicles intact) and after exposure to at least 0.2 MPa (all gas vesicles collapsed). The mean critical collapse pressure was calculated from the pressure-collapse curves [16,44]. The square archaea in the saltern brines did not contribute sufficient turbidity, and therefore this assay could not be performed on the saltern brines.

### Assessment of the vertical movements of square halophilic archaea

Samples of brine or pressurized brine (all gas vesicles collapsed) were placed in a Petroff-Hauser counting chamber (distance between the slide and coverslip: 20 μm). After periods varying from 1–4 hours, the distribution of cells sinking to the grid of the slide, floating to the coverslip, or suspended in between was examined in the phase microscope by focusing the 40× or 100× objective to different levels [45].

We also incubated saltern brine in 1 liter glass cylinders filled to 1 cm below the top. To avoid generation of convection currents that might cause mixing of the brine [35], the cylinders were filled with a gradient from 100% brine below to 90% brine – 10% distilled water on top. The cylinders were incubated at room temperature in diffuse daylight. After different times (up to 5 days), samples were withdrawn from sampling ports at five sampling ports at different depths and the numbers of prokaryotes were counted as outlined above.

To further test for the ability of the square archaea to float, "accelerated flotation" experiments were performed in which brine samples were centrifuged at room temperature at low speeds so that pressures generated were insufficient to cause collapse of gas vesicles. Portions of brine (10 ml in 15 ml Corex tubes) were centrifuged at 500 rpm in a SS-34 rotor in a Sorvall RC-5B centrifuge. With the bottom of the tube being 9.5 cm from the center of rotation, this yielded a centrifugal force of 26 × *g*. Additional experiments were performed in swing-out rotors (Sorvall HB-4 or Hettich Universal 32 centrifuge – radius from center of rotor to the bottom of the sample 14 cm for both). Similar experiments were performed with brines in which all gas vesicles within the cells had been collapsed by pressurization above 0.2 MPa. After different times of centrifugation (up to 60 h), samples were collected from the upper, the middle and the bottom part of the tube, and the numbers of prokaryotes were counted as above.

## Competing interests

The author(s) declare that they have no competing interests.

## Authors' contributions

AO designed most of the experiments, performed part of the laboratory experiments, analyzed the data, and drafted the manuscript.

N.P., O.S. and N.S. jointly designed some of the experiments, performed most of the field work, much of the experimental work, and data analysis.

Calculations of the physical parameters determining buoyancy were a joint effort of O.S. and A.O.
